# Using vignettes to assess the accuracy and rationale of paramedic decisions on conveyance to the emergency department

**DOI:** 10.29045/14784726.2019.06.4.1.6

**Published:** 2019-06-01

**Authors:** Jamie Miles, Joanne Coster, Richard Jacques

**Affiliations:** Yorkshire Ambulance Service NHS Trust; University of Sheffield; University of Sheffield

**Keywords:** allied health personnel, clinical decision making, emergency medical services

## Abstract

**Introduction:**

Paramedics make important decisions about whether a patient needs transport to hospital, or can be discharged on scene. These decisions require a degree of accuracy, as taking low acuity patients to the emergency department (ED) can support ambulance ramping. In contrast, leaving mid–high acuity patients on scene can lead to incidents and recontact. This study aims to investigate the accuracy of conveyance decisions made by paramedics when looking at real life patient scenarios with known outcomes. It also aims to explore how the paramedic made the decision.

**Methods:**

We undertook a prospective mixed method triangulation design. Six individual patient vignettes were created using linked ambulance and ED data. These were then presented in an online survey to paramedics in Yorkshire. Half the vignettes related to mid–high acuity attendances at the ED and the other half were low acuity. Vignettes were validated by a small expert panel. Participants were asked to determine the appropriate conveyance decision and to explain the rationale behind their decisions using a free-text box.

**Results:**

A total of 143 paramedics undertook the survey and 858 vignettes were completed. There was clear agreement between paramedics for transport decisions (ƙ = 0.63). Overall accuracy was 0.69 (95% CI 0.66–0.73). Paramedics were better at ‘ruling in’ the ED, with sensitivity of 0.89 (95% CI 0.86–0.92). The specificity of ‘ruling out’ the ED was 0.51 (95% CI 0.46–0.56). Text comments were focused on patient safety and risk aversion.

**Discussion:**

Paramedics make accurate conveyance decisions but are more likely to over-convey than under-convey, meaning that while decisions are safe they are not always appropriate. It is important that paramedics feel supported by the service to make safe and confident non-conveyance decisions. Reducing over-conveyance is a potential method of reducing demand in the urgent and emergency care system.

## Introduction

In the past decade, there has been rising pressure from health policy for ambulance services to increase the number of patients discharged on scene, and to support paramedics in making autonomous and appropriate decisions about patient care (Committee of Public Accounts, 2011; Lord Carter of Coles, 2016; NHS England, 2013, 2014; NHS Improvement, 2018). Treat and refer (or discharge) mechanisms have been implemented with the creation of alternative care pathways, and new roles, such as advanced clinical practitioners, have been introduced to meet these challenges. However, previous studies have concentrated on specific patient groups and have not measured the effect on the whole system (Mason et al., 2007; O’Hara, O’Keeffe, Mason, Coster, & Hutchinson, 2012; Snooks et al., 2012, 2014).

While previous research has been undertaken around the issue of paramedic conveyance decision making, the accuracy of decisions has not been addressed fully (Burrell, Noble, & Ridsdale, 2013; Cummins et al., 2013; Ebben et al., 2017; Fraess-Phillips, 2016; Halter et al., 2011; O’Hara et al., 2014). Safeguards to decision making found in-hospital, such as senior clinical advice, feedback loops and availability to reassess the patient, are not standard practice in the pre-hospital environment. This makes the ‘right decision, first time’ ethos hard to establish in practice.

The aim of this study is to determine how paramedics approach conveyance decisions and how accurate these are when compared to the actual patient outcome. The research questions for this study are:

1. How accurately can paramedics in the Yorkshire Ambulance Service NHS Trust identify the most clinically necessary destination of the patient, from the context of being on-scene?
2. What themes appear to be important to paramedics when deciding on a patient destination?

The primary objective was to investigate the accuracy in conveyance decision making by paramedics from the context of being on-scene, and the secondary objective was to explore the rationale behind the decisions made.

## Methods

### Design

This study was a ‘validating quantitative data model’ which allows the concurrent collection of both quantitative and qualitative data, separate initial analysis and supportive final synthesis (Cresswell, Plano Clark, Guttman, & Hanson, 2003). Both types of data were collected using an online survey.

### Selection and description of participants

The study was conducted in the Yorkshire Ambulance Service NHS Trust (YAS). This ambulance service provides 24-hour emergency and urgent care for the Yorkshire and Humber region of England, which encompasses a mix of urban, rural and coastal geography. It has 62 ambulance stations, employs nearly 6000 staff and in 2017–2018 responded to 778,639 incidents. During the study data collection period (July–September 2017), there were 1250 paramedics employed by YAS and thus eligible to complete the study. Demographic information such as length of service and educational and professional experience was captured during participation to assess how representative the sample was.

### Vignette creation

A linked database of patient care records (PCRs) from YAS and all emergency departments (EDs) in the Yorkshire region was used to identify the acuity of each conveyance. During 2014 there was a total of 404,348 ambulance conveyances. Any patient who attended the ED by ambulance but received no investigations or treatments and was discharged with no onward referral was considered low acuity (Lowy, Kohler, & Nicholl, 1994; O’Keeffe, Mason, Jacques, & Nicholl, 2018). By applying this definition, the dataset consisted of 373,921 (92.3%) patients who were medium to high acuity and 31,057 (7.7%) who were low acuity. In this study, it was assumed that a low acuity conveyance was clinically unnecessary as the patient could have been effectively managed in a setting other than the ED. Using the linked data, the six most common AMPDS code triage dispositions were identified (Table 1).

**Table 1.** Randomisation of the clinical necessity of patient cases.

AMPDS code		Vignette number	Conveyance clinically necessary	
			No	Yes
Breathing problems	6	1	Selected	
Convulsions/fitting	12	2	Selected	
Psych/ab. beh/suicide	25	3		Selected
Unconscious	31	4	Selected	
Healthcare practitioner referral	35	5		Selected
111 referral	111	6		Selected

The six dispositions were randomly allocated into two conveyance groups: clinically necessary or clinically unnecessary (Table 1). Six subsets of the linked database were then created to identify relevant cases which the vignettes could be based on. Once appropriate cases were identified, patient information was transposed from the PCR to ensure that an anonymised vignette could be created. Once the vignettes were created, each patient journey was considered by a panel consisting of a professor in emergency medicine, an academic GP and the associate director of paramedic practice in YAS. They were asked a binary question of whether the ED was the correct place for that patient episode. Validation occurred with either unanimity or 2:1 vote. The final vignettes are presented in Supplementary 1.

### Participant survey

Participants were recruited via an advertisement published in the internal communication channels in YAS. The vignettes were presented via an online survey hosted by Google forms and were completed by participants in their own time and from their preferred location.

For each case, respondents were asked to read the vignette and answer two questions. Question 1 asked respondents to identify the most appropriate destination from a hierarchical list:

1. Emergency department
2. Minor injuries unit
3. Referral to GP
4. Referral to pharmacist
5. Suitable for self-care

Question 2 was a short-answer free-text box asking respondents to explain their thoughts on destination choice for qualitative analysis.

Data were collected using the online survey hosted by Google forms and exported into Microsoft Excel for Windows Microsoft Office 2016.

### Statistics

For the quantitative analysis, paramedics were considered to be the independent variable, with patient destination being the dependent. Answers were categorised into four groups to create a contingency table (Table 2), to enable calculation of accuracy, sensitivity and specificity, negative predictive value (NPV) and positive predictive value (PPV). Fleiss’s kappa was calculated to measure the agreement between paramedics in the sample size (Fleiss, Levin, & Paik, 2003).

**Table 2.** Stratification of answers.

**Group 1: True positives**	**Group 2: False negatives**
Answers justifying the conveyance of clinically necessary patients.	Answers justifying the non-conveyance of clinically necessary patients.
**Group 3: True negatives**	**Group 4: False positives**
Answers justifying the non-conveyance of clinically unnecessary patients.	Answers justifying the conveyance of clinically unnecessary patients.

For the qualitative analysis, three themes from existing literature were identified: ‘patient safety’, ‘role confusion’ and ‘fear of litigation’. An additional theme identified in the quantitative data was ‘experience’. Each respondent had shorthand tags attached to their responses that captured their demographics. This allowed for comparisons between specific groups. All free-text answers to survey question 2 were stratified by their decision as per Table 2 prior to analysis. This made it possible to explore whether there were any themes surrounding a correct answer compared to an incorrect one (according to study definitions). A thematic analysis was undertaken on each subset of answers and on subsequent comparison analysis between groups. Data were entered into NVivo for Windows V11 and themes were inputted as nodes. Initial word frequency was analysed before thematic analysis and deductive coding was used to support the themes. Inductive coding was not intended but was permitted during the interpretive phase.

## Results

The survey ran for a total of three months (July–September 2017) and 143 participants provided answers to 858 vignettes. At the time of the study, this represented 11.4% of the population of paramedics in YAS. No participants were lost due to incomplete survey participation. Table 3 describes the characteristics of the respondents. The sample size was insufficient to gain statistically significant results if stratified into sub-groups, so this was not attempted.

**Table 3.** Study population characteristics.

Characteristic	Sub-groups	n (%)
Length of service	1–3 years	40 (27.5)
	4–6 years	27 (19)
	7–9 years	32 (22.5)
	10+ years	44 (31)
Professional level	Paramedic	101 (70.4)
	Sp. paramedic	32 (22.5)
	Adv. paramedic	10 (7)
Professional entry method	IHCD	51 (35.7)
	University	92 (64.3)
Highest academic level	IHCD	17 (11.9)
	Undergraduate	52 (36.4)
	Graduate	44 (30.8)
	PGCert/PGDip	15 (10.5)
	Postgraduate	15 (10.5)
Paramedic Pathfinder tool	Used	31 (21.1)
	Not used	112 (78.9)

Table 4 shows the concordance results of the paramedic respondents versus actual patient disposition. From the proportion of participants who made a correct disposition decision, it is clear that Vignettes 2 and 4 presented the greatest challenge to participants.

**Table 4.** Proportion of correct disposition decisions stratified by vignette type (n = 143).

Vignette number	Conveyance clinically necessary	Correct disposition	Incorrect disposition	Proportion of correct disposition (%)
1	No	136	11	92.5
2	No	48	99	32.7
3	Yes	142	5	96.6
4	No	43	104	29.3
5	Yes	133	14	90.5
6	Yes	124	23	84.4

### Quantitative results

Total accuracy was 0.69 (95% CI 0.66–0.73). Agreement between all paramedics was substantial (Fleiss’s ƙ = 0.63). The 858 observations gave a sensitivity of 0.90 (95% CI 0.87–0.93); sensitivity in this context is measuring how accurate paramedics are at ‘ruling in’ the ED (Table 5). Specificity was 0.49 (95% CI 0.44–0.53); specificity is how accurate paramedics are at ‘ruling out’ the ED. The PPV was 0.64 (95% CI 0.60–0.68) and NPV 0.84 (0.78–0.88). Sub-group analysis can be found in Table 6; there were no statistically significant findings when clustering.

**Table 5.** Contingency square of paramedic conveyance decisions.

Paramedic decision	Conveyance clinically necessary		Total
	Yes	No	
**Yes**	399	227	626
**No**	42	214	256
**Total**	441	441	882

**Table 6.** Sub-group analysis.

Group	Cluster	Q1 accuracy	95% CI	Sensitivity	95% CI	Specificity	95% CI	PPV	NPV	Agreement (ƙ)
**Length of service**	*1–3 years*	0.70	0.63–0.77	0.86	0.79–0.92	0.54	0.45–0.63	0.65	0.79	0.80
	*4–6 years*	0.69	0.61–0.78	0.93	0.87–0.98	0.46	0.35–0.57	0.63	0.86	0.80
	*7–9 years*	0.73	0.66–0.80	0.91	0.85–0.97	0.56	0.45–0.65	0.67	0.86	0.39
	*10*+ *years*	0.68	0.61–0.75	0.89	0.83–0.94	0.48	0.39–0.56	0.63	0.81	0.58
**Professional level**	*Paramedic*	0.70	0.66–0.75	0.89	0.86–0.93	0.52	0.46–0.57	0.65	0.82	0.73
	*Sp. paramedic*	0.69	0.61–0.76	0.89	0.83–0.95	0.49	0.39–0.58	0.63	0.81	0.50
	*Ad. paramedic*	0.68	0.54–0.83	0.87	0.75–0.99	0.50	0.32–0.68	0.63	0.79	0.29
**Entry route**	*IHCD (all)*	0.72	0.67–0.78	0.91	0.86–0.95	0.54	0.46–0.62	0.66	0.85	0.56
	*University (all)*	0.68	0.64–0.73	0.88	0.84–0.92	0.49	0.43–0.55	0.63	0.80	0.67
**Academic level** ^1^	*IHCD*	0.70	0.59–0.80	0.86	0.77–0.96	0.53	0.39–0.67	0.65	0.79	0.54
	*IHCD* + *UG*	0.80	0.69–0.91	0.93	0.84–1.08	0.67	0.50–0.84	0.74	0.91	0.96
	*IHCD* + *G*	0.63	0.48–0.77	0.86	0.75–0.97	0.39	0.23–0.55	0.59	0.74	0.72
	*IHCD* + *P.PG*	0.81	0.66–0.95	1.00	–	0.61	0.39–0.84	0.72	1.00	0.23
	*IHCD* + *PG*	0.78	0.62–0.93	1.00	–	0.56	0.33–0.79	0.69	1.00	0.09
	*UG*	0.70	0.63–0.77	0.87	0.81–0.93	0.53	0.45–0.62	0.65	0.80	0.67
	*G*	0.66	0.58–0.74	0.89	0.82–0.95	0.44	0.34–0.54	0.61	0.79	0.77
	*P.PG*	0.70	0.56–0.85	0.89	0.77–1.01	0.52	0.33–0.71	0.65	0.82	0.14
	*PG*	0.69	0.54–0.83	0.89	0.77–1.01	0.48	0.29–0.67	0.63	0.81	0.88
**Pathfinder** ^2^	*Used*	0.69	0.61–0.78	0.91	0.85–0.97	0.48	0.38–0.51	0.64	0.84	0.51
	*Not used*	0.70	0.66–0.74	0.88	0.85–0.92	0.52	0.46–0.57	0.65	0.81	0.66

^1^IHCD: Institute of Healthcare Development; UG: undergraduate; G: graduate; P.PG: partial postgraduate (e.g. PGDip, PGCert); PG: postgraduate.

^2^Paramedic Pathfinder is a decision support tool used in numerous ambulance services (Newton, Tunn, Moses, Ratcliffe, & Mackway-Jones, 2014).

### Qualitative results

#### True positives

In the survey, there were three opportunities for identifying a true positive patient and 399/441 (90.5%) responses were true positives. These presented themselves as a 37-year-old female with self-harm (Vignette 3), an acutely unwell 64-year-old male (Vignette 5) and an 85-year-old male who had called the ‘111’ telephone service (Vignette 6). Word frequency analysis identified that ‘patient’, ‘need’, ‘requires’ and ‘assessment’ were the most commonly used words. The paramedics made patient-centred references when asked to justify. They were not explicit in mentioning their own viewpoint; but appeared to make empathetic decisions through the ‘patient’s eyes’. The decisions were based upon the mitigation of risk, and were presented as unilateral, unchallenged arguments for not leaving a patient at home. Examples of comments made in this group included:

Unsafe to leave patient at home as further risk of self-harm. (Vignette 2, paramedic no. 88, 7–9 years, DipHE/FDSc)

Pt is not safe to be left alone. Clearly complex pt. A call for help has been made by SOMEONE who has clearly recognised the need for additional help/they are unable to cope. (Vignette 6, paramedic no. 141, 7–9 years, IHCD, Ma/MSc)

Paramedics in this group referred to the safety of the patients more frequently than in the other three groups. They were often making references to safety as the justification to convey. Fear of litigation appeared in a subtle form as paramedics would not always reference the clinical need to convey but, rather, context-related factors, such as ‘ruling out’ the scene as an appropriate place. Role confusion did not appear prevalent in this group.

#### False negatives

In 42/441 (9.5%) cases, paramedics incorrectly decided not to convey the patients in Vignettes 3, 5 and 6. Word count analysis showed similar words to the first group, with the addition of ‘care’ and ‘possible’. This group had the largest number of uncertain modal verbs; in particular the word ‘possible’. This was used as a prefix to a statement of diagnostic conjecture. For example:

possibly needs pacemaker if concerns bradycardia is new and due to conduction abnormality. (Vignette 6, advanced paramedic no. 27, 10+ years, PGDip/PGCert)

Despite electing not to convey the patient, little mention was made of self-care, and most stated that they would refer the patient to the GP when the ED was deemed too extreme. This can be evidenced with the following quotes:

Would prefer to see return to respite but would depend on level and severity of D&V/dehydration and if flutter was related. (Vignette 6, paramedic no. 89, 7–9 years, IHCD)

If atrial flutter and bradycardia is known then this is possibly suitable for bed bureau admission for monitoring of D&V. (Vignette 6, paramedic no. 67, 7–9 years, BSc/BA)

It appears that paramedics were ruling in alternative care as opposed to ruling out the ED. For Vignette 5, paramedics challenged the GP decision for conveyance to the ED, and made suggestions about utilising an alternative pathway. Paramedics in this group made frequent references to the GP already making the decision in Vignette 5. This may indicate fear of litigation as paramedics have negated their own clinical assessment and judgement as a perceived senior clinician has taken on accountability of care. Paramedics in this group made reassuring statements that indicate they were conscious of the safety of patients when making the decisions.

#### True negatives

There were 214/441 (48.6%) correct responses by paramedics who identified that a clinically unnecessary ED attendance could be avoided. Three vignettes gave the paramedics an opportunity to output a true negative. These were a 23-year-old female with breathing difficulty (Vignette 1), a 19-year-old female who passed out (Vignette 2) and a 21-year-old female who was unconscious (Vignette 4). Similar word frequency was noted in this group compared to the previous two, with the addition of the word ‘normal’. Paramedics in this group commonly referred to the normality of the patient as opposed to an extreme of condition. Their language appeared to justify not conveying the patient by emphasising the normality of their current presentation:

all observations are now within normal ranges, paraesthesia now absent points to anxiety attack. (Vignette 1, paramedic, 1–3 years, BSc/BA)

This is a known neurological problem for this patient. She is aware of it and it is normally self limiting. I would have a conversation with the ECPs regarding this patient and take their advice. (Vignette 2, paramedic, 1–3 years, DipHE/FDSc)

Patient had fainted but ECG is normal and obs have stabilised. I do not think this requires further emergency treatment. (Vignette 4, advanced paramedic, 4–6 years, IHCD, BSc, MSc)

This is an inductive theme that has not been identified previously in the literature. The true negative group appears to be ruling out the ED as an appropriate place. The true positive answers seemed to make attempts to rule out non-conveyance instead. This is in contrast to the false positive and false negative groups, which appeared to use language that ruled in. This could link to experience.

#### False positives

There were 227/442 (51.4%) responses where conveyance to ED was incorrectly chosen by paramedics for Vignettes 1, 2 and/or 4. Word count analysis highlighted the presence of different words compared to the other groups. The most common words were ‘episode’, ‘need’, ‘causing’ and ‘investigation’. The word ‘episode’ was referenced by 57 participants, especially in Vignette 2 (19-year-old female unconscious). Paramedics were deciding to rule the ED in, for example:

Although similar to previous episodes, this one differs in that it has not resolved spontaneously. This may give window for tests/scans which may be revealing. (Vignette 4, paramedic no. 80, 7–9 years, IHCD, DipHE/FDSc)

This episode is still occurring and more likely having localised seizures. Requires meds and CT scan and bloods. (Vignette 4, specialist paramedic no. 63, 4–6 years, BSc/BA)

This could link to experience or fear of litigation as they are justifying their decision by describing the possible investigations and treatments the patient could need, and that they cannot directly deliver. The paramedics could also have been confused as to the role of the ED and what investigations and treatment patients were likely to receive at the ED.

## Discussion

Paramedics that took part in this study demonstrated a high level of accuracy in their transport decisions to the ED. However, high sensitivity but low specificity suggests that over-conveyance is a problem. Cummins et al. (2013) used concordance compared to ED physician in their study of whether advanced paramedics in Ireland could diagnose and predict admission. They found an overall concordance of 70% for diagnosis, which is very similar to the clinical disposition accuracy of this study (69.5%). The outcome measures for the Cummins study were different to this study, as they examined concordance with an ED physician compared to actual patient outcome in this study. Concordance in this study was aggregated across vignettes. When exploring individual vignette concordance, two were significantly lower than in others (Vignettes 2 and 4). These had clinical symptoms which make decisions less clear, such as dizziness and numbness. Studies have explored ambulance presentations of acute-on-chronic neurological conditions such as epilepsy. They have found that even though the ambulance had been called to a seizure, if the patient is transported they rarely receive a clinical benefit at ED (Dickson et al., 2017; Dickson, Taylor, Shewan, Grünewald, & Reuber, 2016). Other outcome measures that have been utilised include the accuracy of a tool. Newton, Tunn, Moses, Ratcliffe and Mackway-Jones (2014) examined the accuracy of the Paramedic Pathfinder tool, a decision support tool used by multiple ambulance services in the United Kingdom. Their study used a ‘gold standard’ of expert clinicians to review the decision outputs for actual patients. They found the tool had a concordance between ambulance clinician and the gold standard of 80.5%, with a sensitivity of 94.8% and specificity of 57.9% – an improved result over the results in this study.

The results seen in this study support previous qualitative research findings that system factors, such as patient safety and fear of litigation, are key in non-conveyance decision making. This directly contrasts with recent healthcare policy which advocates that paramedics be the final arbiters of care on-scene (Lord Carter of Coles, 2016; NHS Improvement, 2018).

Finally, this study has identified a new theme of paramedics using the ‘normality’ of the patient as an anchor from which to make a reference regarding their current condition. This means they compare a patient’s current condition to what is normal for the patient and use this as a decision-making tool. This would need exploring further in subsequent research.

### Limitations

This study was theoretical as opposed to pragmatic in design. Although the vignettes were based on actual patient encounters, the participants completed the study in front of an electronic device (away from a patient). Future studies exploring this topic should observe real-time decisions in practice. Another limitation of the methodology was the definition. Despite the selection being based on a double validated definition and checked by a small panel of experts, it simplifies decisions to a binary form. For example, there are likely to be true positive patients that could have had care provided in the community. Equally, there could have been non-conveyed patients who subsequently saw the ambulance service shortly after discharge. The study methodology used here could not discriminate patients beyond the definition. In addition, the choice of qualitative methodology was based on open-ended questions in a survey. While the results are interesting, a separate study with superior methodology is needed to validate the findings.

The sampling method could result in selection bias; however, the internal communication gave every staff member an equal opportunity to take part. Another way to overcome limitations in a future study would be to employ a more purposive selection of patient cases.

In this study, the vignettes were randomly selected and screened. However, this resulted in a disproportionate number of young female patients, and subsequently a bias. For future studies, it might be more appropriate to screen by the level of information provided. This would allow an analysis of how paramedics make different decisions based on the information they are presented with.

## Conclusion

In this study, paramedics made accurate conveyance decisions but were better at ruling the ED in, compared to ruling it out. The high proportion of decisions to convey clinically unnecessary patients indicates risk mitigation and a focus on patient safety. This is supported by qualitative analysis that demonstrated through the different sub-groups that paramedics focused on patient safety and fear of litigation.

## Author contributions

JM: Research design, data collection, data analysis, manuscript lead.

JC: Research design, literature sourcing, qualitative results (second reviewer), manuscript editor.

RJ: Research design, statistical literature, statistical support, quantitative results (second reviewer), manuscript editor.

## Conflict of interest

None declared.

## Ethics

The study was approved by the University of Sheffield ethics review panel.

## Funding

This study was undertaken as part of a Master’s in Clinical Research. The funding for the dissertation was provided partly by Yorkshire Ambulance Service NHS Trust and partly by the NIHR CLAHRC Yorkshire and Humber (www.clahrc-yh.nihr.ac.uk). The views expressed are those of the authors, and not necessarily those of the NHS, NIHR or Department of Health.
